# Fucosylated but Not Sialylated Milk Oligosaccharides Diminish Colon Motor Contractions

**DOI:** 10.1371/journal.pone.0076236

**Published:** 2013-10-02

**Authors:** John Bienenstock, Rachael H. Buck, Hawley Linke, Paul Forsythe, Andrew M. Stanisz, Wolfgang A. Kunze

**Affiliations:** 1 McMaster Brain-Body Institute at St Joseph’s Healthcare, Hamilton, Ontario, Canada; 2 Abbott Nutrition, a Division of Abbott Laboratories, Columbus, Ohio, United States of America; 3 Department of Pathology and Molecular Medicine, McMaster University, Hamilton, Ontario, Canada; 4 Department of Medicine, McMaster University, Hamilton, Ontario, Canada; 5 Firestone Institute for Respiratory Health, Hamilton, Ontario, Canada; 6 Department of Psychiatry and Behavioural Neurosciences, McMaster University, Hamilton, Ontario, Canada; 7 Farncombe Family Digestive Health Institute, Hamilton, Ontario, Canada; Cincinnati Children's Hospital Medical Center, University of Cincinnati College of Medicine, United States of America

## Abstract

Human milk oligosaccharides (HMO) are being studied by different groups exploring a broad range of potential beneficial effects to the breastfed infant. Many of these effects have been attributed to a growth promotion effect on certain gut organisms such as bifidobacteria. Additionally, evidence indicates that HMO are able to directly promote positive changes in gut epithelium and immune responses under certain conditions. This study utilizes a standardized *ex vivo* murine colon preparation to examine the effects of sialylated, fucosylated and other HMO on gut motor contractions. Only the fucosylated molecules, 2’FL and 3’FL, decreased contractility in a concentration dependent fashion. On the basis of IC_50_ determinations 3’FL was greater than 2 times more effective than 2’FL. The HMO 3’SL and 6’SL, lacto-N-neotetraose (LNnT), and galactooligosaccharides (GOS) elicited no effects. Lactose was used as a negative control. Fucosylation seems to underlie this functional regulation of gut contractility by oligosaccharides, and L-fucose, while it was also capable of reducing contractility, was substantially less effective than 3’FL and 2’FL. These results suggest that specific HMO are unlikely to be having these effects via bifidogenesis, but though direct action on neuronally dependent gut migrating motor complexes is likely and fucosylation is important in providing this function, we cannot conclusively shown that this is not indirectly mediated. Furthermore they support the possibility that fucosylated sugars and fucose might be useful as therapeutic or preventative adjuncts in disorders of gut motility, and possibly also have beneficial central nervous system effects.

## Introduction

Human milk oligosaccharides (HMO) constitute a repertoire of more than 100 soluble glycan structures. Their potential beneficial effects for the breastfed infant have been studied by several researchers but only one clinical trial has been reported [1-3]. The possibility to examine biological effects of HMO has increased due to technical advances which now offer for exploration previously unavailable synthetic carbohydrates. Milk oligosaccharide content varies amongst different mammals, however, they are a major molecular class in human breast milk while other species have limited and less diverse repertoires. For example, bovine milk has been shown to contain less complex, diverse and generally less abundant structures than human milk [4] and it has been generally agreed that fucosylated oligosaccharides such as 2’FL are not present. However a recent paper by Sundekilde, Barile, Meyrand, Poulsen, and Larsen 2012 [5] have shown for the first time the presence, albeit at low concentration, of some more polymerized fucosylated milk glycans. Accordingly, infant formulas and nutritional supplements derived from bovine milk lack the glycan diversity present in mother’s milk.

The observation that the feces of breastfed infants differed in their microbial content from formula fed infants supported early attempts to explain certain of the perceived advantages of breastfeeding (for review see Bode [1]). Several recent studies have identified the ability of specific, generally desirable, gut microbiota (e.g., bifidobacteria) to metabolize both sialylated and fucosylated HMO and to flourish when HMO are available as fermentation substrates *in vitro* and *in vivo* [6-8]. Additionally, Chichlowski, et al, have demonstrated HMO- associated effects on colonic cell lines that are mediated via bifidobacteria [9]. Nevertheless, it is still broadly assumed that HMO are advantageous to the host as a result of indirect action of different groups of gut bacteria, which include bifidobacteria and lactic acid bacteria, whose growth and activity they promote [10]. These observations have led to the introduction of the term prebiotics which are said to be beneficial to the host through this action (9).

However HMO can have direct effects on intestinal epithelial structure and function [11,12], and can interfere with the adhesion of infectious bacteria such as *Campylobacter jejuni* [13], HIV [14] and protozoan parasites such as *Entamoeba histolytica* [15]. Effects of HMO on the immune system have been shown by studies with both mouse and human T-lymphocytes and dendritic cells, and are mediated by interactions with cell surface C-type lectins such as P- and E-selectins, and DC-SIGN. The latter specifically binds high mannose and/or fucose-containing glycans [1]. We have found no evidence in the literature for HMO effects upon gut contractility.

Certain lactobacilli have direct effects within minutes on the enteric nervous system and on neuronally dependent gut motor contractions [16-18]. The amplitude of such peristaltic contractions of small and large intestines induced by increased pressure was reduced within minutes of luminal administration of a specific *Lactobacillus* strain (JB-1) but not by a *Lactobacillus salivarius* [16].

In a subsequent recent publication we showed that a capsular exopolysaccharide of another symbiotic but Gram negative bacteria (*Bacteroides fragilis*) in the gut lumen has similar effects to the parent bacteria and JB-1. This suggests the possibility that other glycans may also be functionally effective in this physiological system. We have therefore turned our attention to the examination of HMO on neuronally dependent colonic contractions [18,19].

We surmised that these effects would be easier to observe and measure in the absence of confounding factors introduced by the nutritional context and processing by the gut microbiome that may occur after oral administration.

## Materials and Methods

Endotoxin free Krebs buffer was constituted as previously described [16]. Test sugars were obtained as a gift from Abbott Nutrition (Columbus, OH, USA). Purity and endotoxin concentrations are summarized in Table **1**. HMO "purity" was established by high performance ion chromatography with pulsed amperometric detection (IC-PAD) using relative peak area comparisons. Moisture content was determined separately using the Karl Fischer method for moisture determination. 3’-Sialyllactose (3’ SL), 6’Sialyllactose (6’SL) and 2’Fucosylactose (2’FL) were all derived from bacterial synthesis. 3’-Fucosylactose (3’ FL) was chemically synthesized and lacto-N-neoTetraose (LNnT) was synthesized from a yeast fermentation system and purified by crystallization [3]. The galactooligosaccharides (GOS) preparation consisted of galactose (1.23%), glucose (15.8%), lactose (9.26%), 48.4% GOS and 25.3% water. L-fucose (catalogue number F2252) and β-lactose were obtained from Sigma (St Louis, MO, USA). Endotoxin levels were estimated by limulus assay (Limulus Amebocyte Lysate (LAL) QCL-1000, Lonza catalogue number 50-647U, Wilmington, MA, USA) and lipopolysaccharide (LPS) 500,000 EU/mg from Sigma (catalogue number L2637, St. Louis, MO, USA).

 Adult male Swiss Webster mice were obtained from Charles River (Raleigh, NC, USA). Handling of animals and all experimental procedures were conducted in accordance with the guidelines of The Canadian Council on Animal Care and approved by the McMaster University Animal Research Ethics Board.

### Organ bath intraluminal pressure recordings:

The method we used has recently been published [18] and is similar to that described by us [16] for jejunal motility. It follows the technique described by Keating et al [20] for measurements of colon. Briefly, mouse distal colon 4cm segments were excised and flushed with Krebs buffer under a 2 hPa gravity pressure head. Designated “oral” and “anal” ends of each segment were cannulated, mounted in a 20mL organ bath chamber and submerged in oxygenated Krebs. The lumen was gravity perfused at 0.5mL.min^-1^ with carbogen-gassed Krebs (95% O_2_ and 5% CO_2_ at room temp). The organ chamber (serosal compartment) was perfused with the same pre-warmed (34°C) Krebs buffer at 5mL.min^-1^. This temperature was chosen since it preserves stable gut function for up to 2 hours. At the beginning of the experiment, intraluminal pressure of 5 hPa was obtained by adjusting the heights of inflow and outflow tubes, and recordings were made at this pressure. Test materials were applied by switching the oral luminal inflow from Krebs to Krebs plus test substance by closing and opening the appropriate stopcocks.

Recordings were analyzed off-line using the Clampfit module of PClamp 9 software (Molecular Devices) as previously described for jejunal motility analysis. Intraluminal pressure changes were measured at the midpoint of the longitudinal axis of the gut segment and the pressure signals were amplified, digitized, and stored on a PC computer. Peak pressures (PPr) for individual migrating motor complexes (MMC) were measured using the cursors in Clampfit as the difference between baseline and the maximal pressure reached during the PPr. Control PPr was calculated as the average from at least 6 successive motor complexes with Krebs perfusing the lumen just before the intraluminal perfusate was switched to one containing an HMO. Then, a further 6 PPr were measured between 15 to 30 min after beginning the HMO application and after which the effects on PPr had plateaued. The latter 6 measurements were averaged to provide the “after” PPr value in the paired “before and after” experiments.

For each experiment HMO were only applied once at a particular concentration because when a given HMO altered PPr the effect did not fully wash out even after switching the luminal perfusate to Krebs buffer for 2 h. Responses each single experiment were displayed as connected lines rather than bar graphs to emphasize the before and after nature of the experimental design. Concentration-response relations were plotted using GraphPad Prism 6.0 (GraphPad Software, San Diego, CA, USA) from the pooled data of individual before and after experiments. Log (HMO)-PPr plots were fitted by a 3-point logistic (Hill) equation of the form Y = bottom + (top - bottom)/(1 + 10^X - log IC50^), where IC50 is the concentration of the HMO that produces 50% inhibition.

#### Video Recordings

We also employed a recently developed video imaging system to record peristalsis of colonic motor contractions [18] to confirm the results we obtained in peak pressure recordings. These allow an analysis in real time of MMC and relaxation of the gut wall. These can be converted to colour in a heat map format, and additionally provide the opportunity to quantitate frequency of contractions and their time course, thus allowing calculations of velocity.

#### Statistics

All statistics were calculated using GraphPad and descriptive statistics given as means +/- SE, and significance tests made using the Wilcoxon matched-pairs (before/after) signed rank test. The statistically discernible difference for tests of significance was set at P = 0.05; all tests were 2-tailed. Significance is indicated on graphs using conventional markers: *P = < 0.05; ns: P > 0.05

## Results

**Figure 1:**
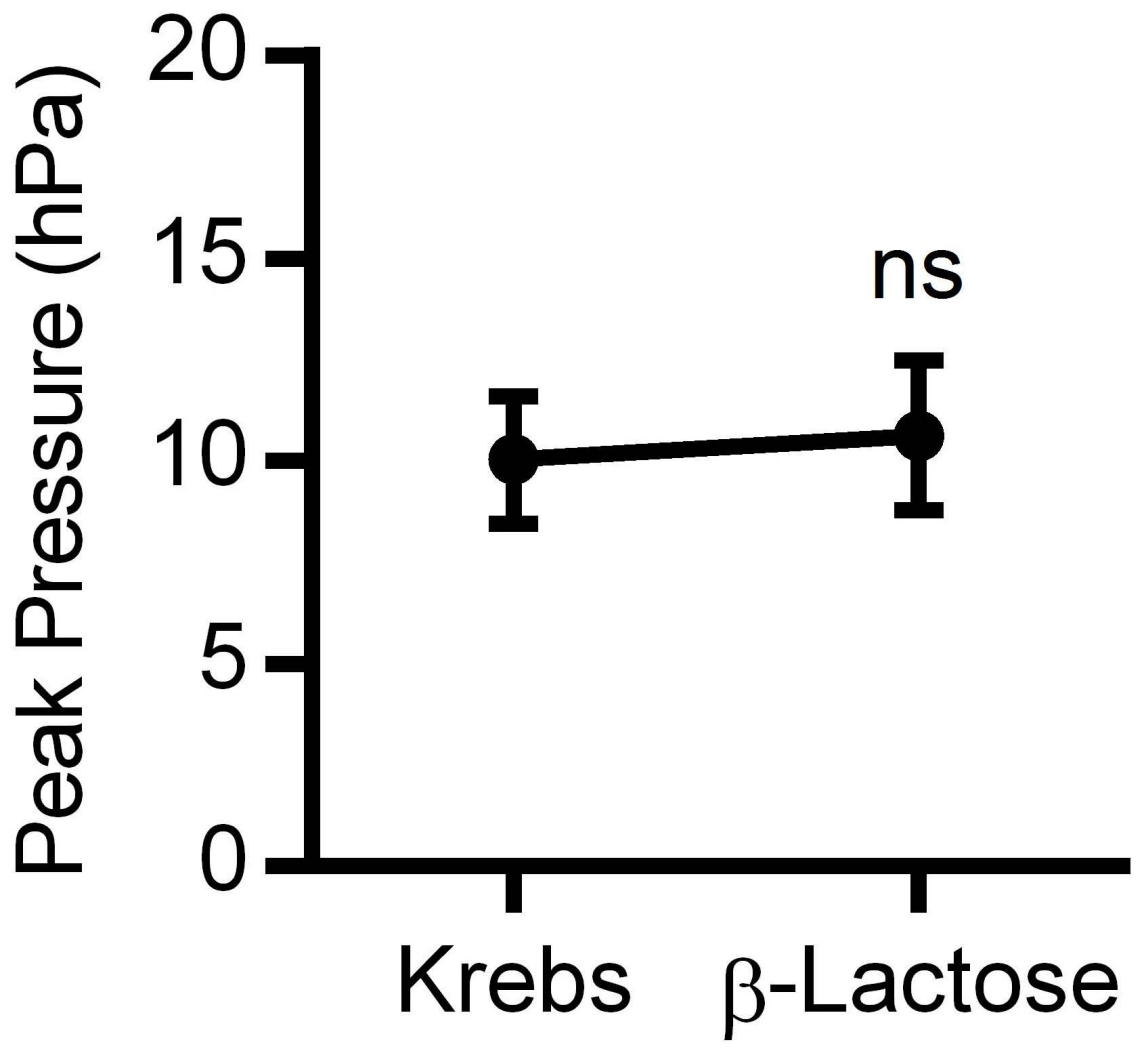
Effects of lactose on peak pressures in colon motility experiments. Lack of effect of β-Lactose (1mg/mL) on peak preasures of migrating motor complexes in 'before and after' experiments (n=6). ns=not significant.

Lactose did not alter PPr (P = 0.8, *n* = 6) at 1mg/mL or over a concentration range 0.5-3.0mg/mL and was thus used as a negative control when compared to Krebs buffer by itself as shown in Figure 1, and was used as such throughout the study.

**Table 1:** Characteristics of HMO and Monosaccharides

HMO and Monosaccharides	% Purity	Endotoxin Concentration EU/mg
3’Sialyllactose (3’SL)	97.1%	0.015
6’Sialyllactose (6’SL)	96.6%	0.496
2’-Fucosylactose (2’FL)	95.3%	0.375
3’-Fucosylactose (3’FL)	99.7%	Not Tested
Lacto-N-neotetraose (LNnT)	95.2%	0.34
Galactooligosaccharides (GOS)	48.4%	0.58
L-Fucose	99.0%	Not Tested
Lactose	99.0%	0.002

**Figure 2:**
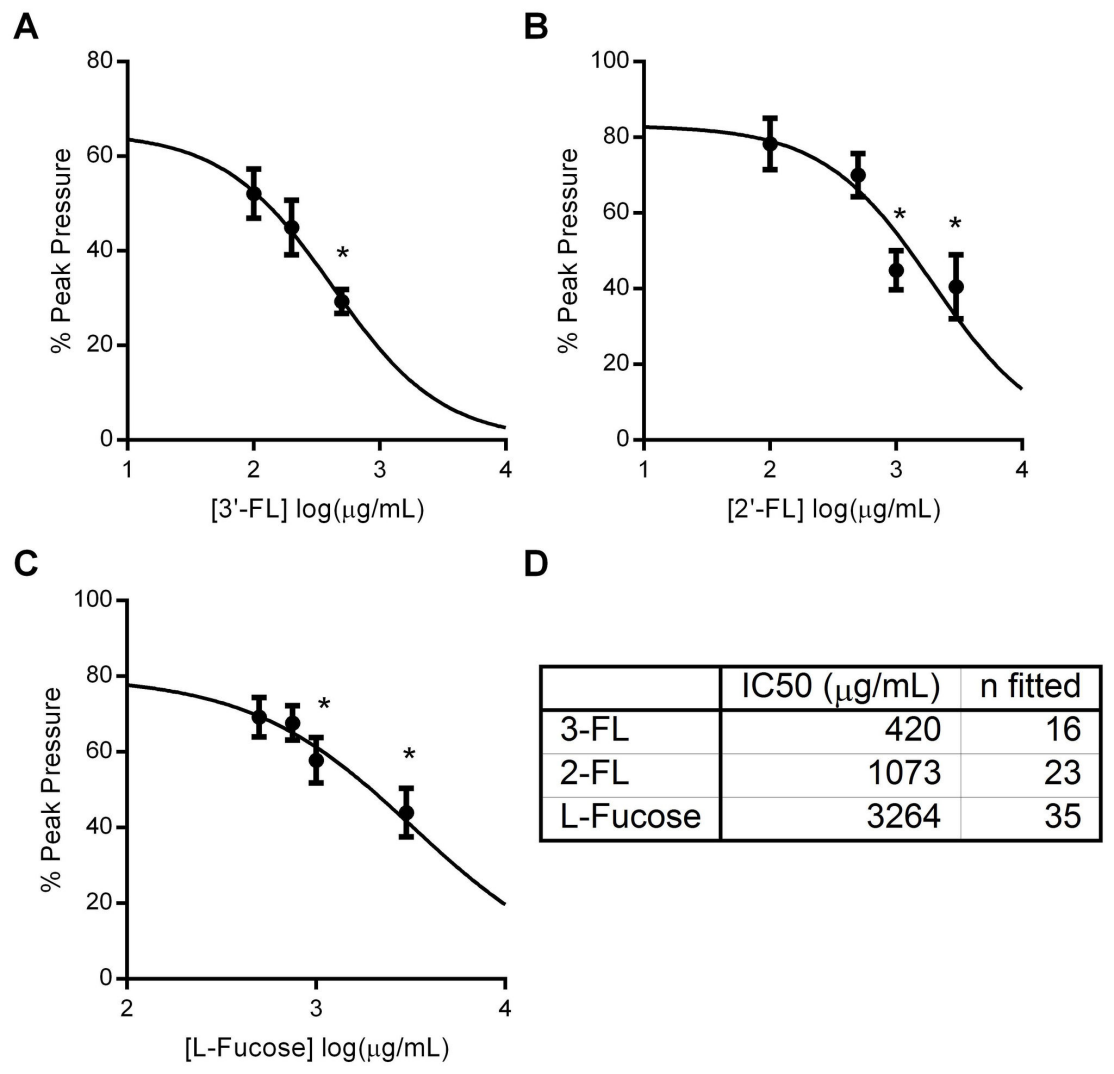
Concentration response curves for effects of fucosylated HMO. Effects of 3’FL (A), 2’FL (B) and L-Fucose (C) on peak pressure. Inset table indicates the number of points fitted to the curve and IC_50_ values. * p values <0.05.

The recorded propagated MMC were neuronally dependent since they were inhibited entirely by prior addition of tetrodotoxin (TTX), a specific neuron inhibitor (Figure S1), and as also shown in this figure, TTX inhibited the diminished contractions after the addition of HMO in keeping with our previous observations with jejunal preparations [16]. Of all the glycans tested (see Table 1 for list), only two (2’FL and 3’FL) demonstrated effects on PPr. Figure 2 shows the magnitude of the responses for the two effective HMOs tested (Figure 2 A& B) as well as the monosaccharide L-Fucose(Figure 2C). The onset of PPr reduction ranged from 5-15 min and did not wash out after switching the intraluminal perfusate back to one containing only Krebs buffer for up to 2 h. The IC_50_ for 3’FL was 420µg/mL, for 2’FL 1073µg/mL (Figure 2A and B) and 3264 µg/mL for L-Fucose. Since the effects were only observed with glycans containing fucose, we examined if L-fucose alone could modulate PPr of motor complexes in this system. As shown in Figure 2C, fucose also diminished PPr, but to a significantly lesser extent. The L-fucose effect was exhibited at a higher concentrations (IC_50_ =3264 µg/mL) than required for the fucosylated HMO. These results are summarized in Figure 2 inset table.

**Figure 3:**
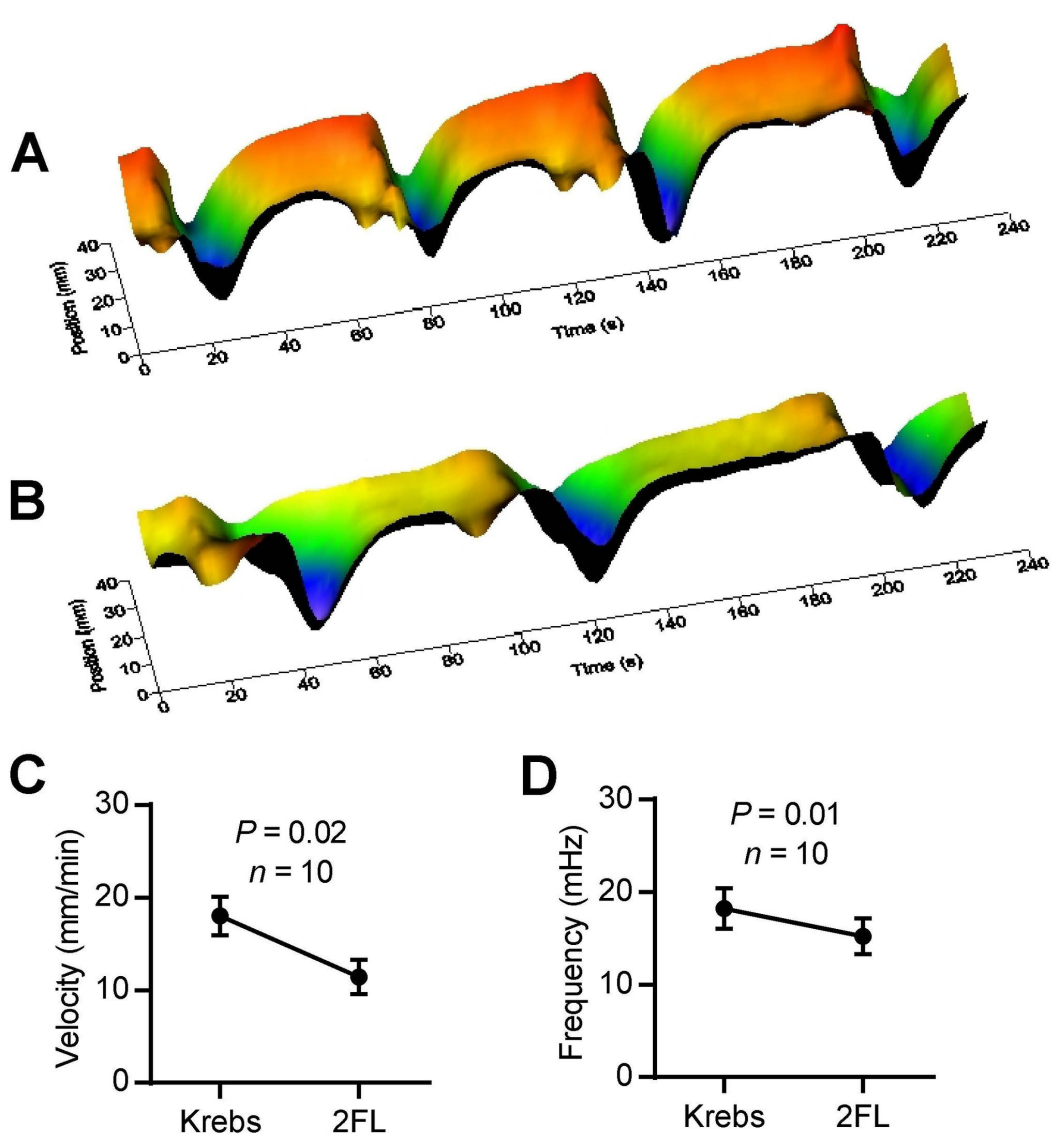
Effects of 0.5 mg/mL intraluminal 2’FL on colon motility. Heat maps derived from black/white spatio-temporal video recordings of colon motility. (A) Shows regular migrating motor complexes (MMCs) during control recording. (B) Addition of 2’FL to the lumen decreased both the slope (MMC **velocity**) of the valleys and MMC frequency. (C, D) Summary statistics of before and after experiments showing that 2’FL significantly reduced both MMC velocity (C) and frequency (D).

We wished to confirm these results using a recently developed video imaging system [18] and applied this to testing the effects of 2’FL and fucose. As seen in the heat map of the effects of 2’FL at 0.5mg/mL (Figure 3) there is a statistically significant decrease in both frequency of contractions and their velocity in before and after experiments.

**Figure 4:**
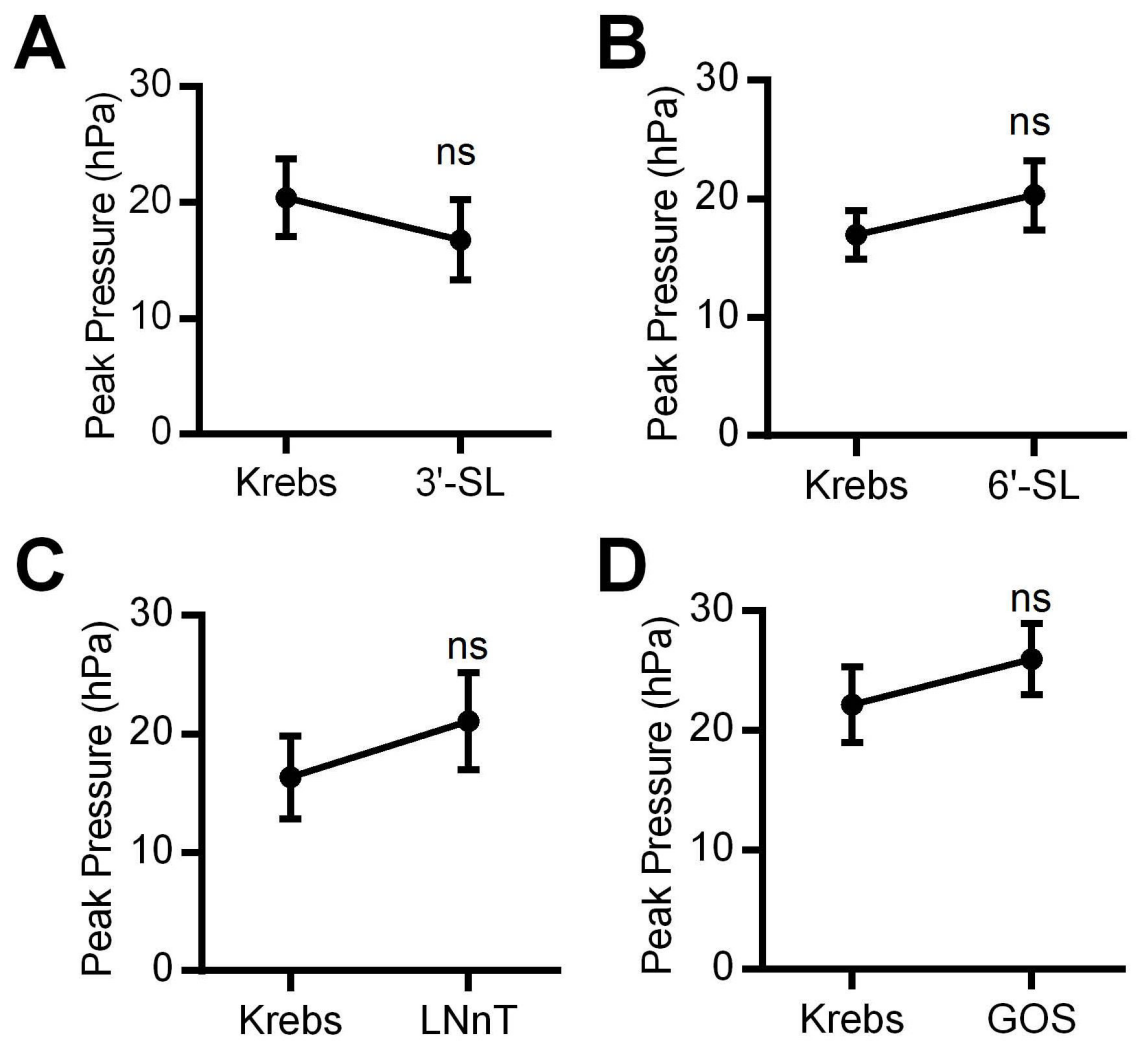
Before and after results of effects of non-fucosylated HMO. Lack of effects of 3’ SL (A), 6’SL (B), LNnT (C) and GOS (D). n=>6/oligosaccharide tested. ns= not significant. Concentrations of all HMO shown at 1mg/mL for comparison purposes. None had any effect on motility up to 5mg/mL.

The results from experiments when sialylated HMO 3’ SL (P = 0.8, *n* = 6) and 6’SL (P = 0.6, *n* =6), as well as LNnT (P = 0.2, *n* = 6) and GOS (P = 0.2, *n* = 6) were applied for up to 1 h are shown in Figure 4. The highest concentration evaluated for any of these HMO was 5mg/mL and no significant effects on PPr were observed at any lower concentration (>0.5mg/mL). Since the glycan preparations were all contaminated with LPS, albeit at minimal concentrations, we tested the effects of its addition to the luminal perfusion fluid, at 100 and 500 EU/mL, concentrations, significantly in excess of the endotoxin levels recorded for the oligosaccharide preparations (as indicated in Table 1). LPS demonstrated no effects at either concentration (data not shown).

## Discussion

The health benefits of breastfeeding for the infant are well documented. Attempts to delineate roles for the myriad components putatively responsible for these effects have been influenced by the fact that while infant formulas differ substantially from breast milk, efforts are continually being made to improve their composition. One example has been supplementation with non-HMO glycans such as GOS and fructo-oligosaccharides (FOS) which themselves have significant positive effects on immune responses [21].

HMO are widely thought to provide a number of health benefits through their activity and interactions with immune and endocrine systems [1]. They are resistant to gastric acid and the small intestine environment and are mostly unaltered by the time they reach the large intestine [22-26], however this appears to depend on the age of the infant, maturity of gut adaptation, and the concomitant ingestion of alternative nutrients [25,26]. Evidence of growth promoting effects on bifidobacteria and lactic acid bacteria and other alterations to the colonic microbiome suggest a causal relationship with health benefits [1,10]. However specific HMO have clear direct effects on host tissues and their component cells such as epithelium [11,12]. Our observations in the *ex vivo* mouse colon model of peristalsis clearly show that simple fucosylated molecules have immediate effects within 5-15 minutes upon colonic neuronally dependent smooth muscle contractions. The further observation that L-fucose itself had similar functional effects (decreased amplitude of intraluminal filling pressure induced migrating motor complexes), suggests a potential role for this monosaccharide and other fucosylated molecules such as 2’FL and 3’FL, in the regulation of gut motility. On a relative concentration basis, 2’FL is almost three times more active than L-fucose, and 3’FL is additionally greater than two times more effective than 2’FL. Interestingly, while 3’FL is a normal constituent of mouse milk, 2’FL is not [27]. Mouse intestine, therefore, may not be as functionally adapted to respond to an oligosaccharide, which is not normally delivered in maternal milk. It should also be noted that unlike the neutral 2’ and 3’ fucosylactoses, the neutral HMO LNnT and the acidic sialylactose examples, 3’ SL and 6’SL showed no demonstrable effect in the motility model. Taken together these results may point to at least two mechanistic hypotheses; on the one hand, the effect of free fucose, albeit small, suggests that the monosaccharide may be limited in intestinal tissue and that its sudden availability fosters the local synthesis of fucosylated glycoconjugates that in turn attenuate neuronal activity. This suggestion would apply more to the *in vivo* situation than the *ex vivo* model we have used. However, the fact that specific fucosylated glycoconjugates such as 2’FL and 3’FL are able to elicit a substantial reduction of gut motility suggests a specific interaction of fucose and/or fucose residues with tissue receptors that in turn regulate gut motility.

The best interpretation of the demonstrated modulation of neuronally dependent migrating motor complexes predicts that fucosylated molecules could demonstrate anti-nociceptive activity as well. Large amplitude motor complexes appear to be essential for the perception of visceral pain [28], so that a reduction in amplitude as demonstrated by fucosylated HMO ought to moderate the nociceptive stimulus. The further exploration of the potential use of these particular HMO in conditions associated with disordered motility and gut pain, such as functional gut disorders and infantile colic, appears warranted.

The mechanism(s) whereby certain simple sugar components of breast milk may directly influence the migrating motor complexes is not known. “Bifidogenesis” is improbable because preparation of the colon segments removes most non-adherent contents. The rapid response (5-15 min) of gut musculature to exposure to the luminally perfused oligosaccharides suggests that the very limited number of existing bacteria still present in adherent mucus, are extremely unlikely to have been responsible for the motility effects. While we can rule out bifidogenesis we cannot rule out indirect effects from the few bacteria present in mucus. Furthermore, we have recently shown that a complex glycan, a bacterial exopolysaccharide, was neuronally active within seconds of placement into the lumen of an intact gut segment [17]. Again, this evidence does not rule out possible indirect effects through primary effects on epithelium or immune cells below the epithelium. However, both simple and complex glycans can influence the ENS almost immediately. While HMO do not appear to be absorbed across the intestinal epithelium intact *in vivo*, experiments with monolayers of epithelial cell lines have shown that both acidic and neutral HMO can be transported [29,30]. Neutral HMO are transported both transcellularly and via paracellular pathways [30], leaving open the possibility that the effects seen are indirectly mediated.

While neurons do express glycan receptors which do not bind fucose, such as galectins [31] and TLR [32-34], the presence of DC-SIGN which binds fucose and mannose with equal high affinity has not been reported. Free fucose has been shown to have a direct effect on differentiated Caco-2 epithelial cells, which appear unable to metabolize the sugar, although it promotes a TLR-2-like signaling pathway [35]. Alternatively, simple diffusion or transport of the oligosaccharides themselves across the epithelium may promote neuronal interaction since axonal terminals are known to be present in lateral intercellular spaces and immediately below the basal surfaces of the epithelium. Fucose itself has been described in the older literature as being highly immunoregulatory both *in vivo* and *in vitro* [36,37] and such actions have also been recorded for 2’FL (Sotgiu et al 2006) [38]. It is therefore plausible to postulate that it may be exerting similar direct effects on the enteric nervous system as it does on epithelial and immune cells.

The use of a mouse colon segment as a model for potential human application may be intuitively questioned. However, this assay has been validated as a potential screening method for pharmacological agents with known or potential therapeutic effects on human gut [20]. The authors’ conclusions are also reflected in the title of the paper: “The validation of an *in vitro* colonic motility assay as a biomarker for gastrointestinal adverse reactions”. The motor complexes which we record, are entirely neuronally dependent, since they are completely abolished by the specific sodium channel blocker and neurotoxin TTX (Figure S1).

Fucosylated molecules regulate the synapse function and development as well as neuronal morphology in primary hippocampal neuron culture [39]. They are also involved in cognitive aspects of brain function such as task-specific learning and long-term potentiation [40-42]. The concentrations of specific HMO in breast milk vary according to secretor status and time after parturition [43]. In the first 3 months of lactation the highest concentration of 2’FL in 12 donors approximated 3g/L [44] which thereafter declined to a mean of 1.2g/L. The concentrations of HMO which we have tested therefore fall into the likely physiological ranges occurring in the intestinal lumen in breast-fed infants. Given the possibility that small amounts of HMO may be transported or translocated intact across the intestinal epithelium, it is possible that in infancy, dietary fucosylated oligosaccharides and their degradation products such as fucose, may play an important role in the development and robust function of the central and enteric nervous systems.

Our results support further investigations of fucose, and fucosylated carbohydrates such as 2’FL and 3’FL as specific adjuncts to improve function of the enteric nervous system, and the preventative or therapeutic treatment of disorders involving gut nociception, contractility and motility. These suggestions are supported for 2’FL by our observations with video recordings showing decreased frequency, reduction of amplitude and velocity of colonic motor contractions. Since the effects of fucosylated oligosaccharides clearly occur through interactions, directly or indirectly with the ENS, we speculate that they could well also be exerting a positive effect on the brain via the vagus nerve [45,46] in supporting cognition and memory [47,48].

## Supporting Information

Figure S1 **Lack of direct effect of 0.5 mg/mL 2’FL on colon muscle contractions when neurons are silenced.** (A) Representative intraluminal pressure trace showing that addition of the specific neurotoxin, tetrodotoxin (TTX) at 0.3µM to the solution perfusing the gut segments rapidly abolished MMC pressure waves, leaving only contractile (ripples) that are entirely dependent on the musculature. Intraluminal 2’FL was applied after 15 minute recording with TTX and this had no effect on contractility in the absence of neural activity. (B) Summary statistics for 6 experiments showing that 2’FL had no statistically significant effect on the frequency with which ripples occurred.
(TIFF)
